# Kynurenine pathway metabolites are increased in inflammatory depression and decrease with omega-3 treatment

**DOI:** 10.1016/j.bbih.2026.101221

**Published:** 2026-03-25

**Authors:** Jesper Lindahl, Gustav Söderberg Veibäck, Klara Suneson, Wilma Blanking, Johanna Tjernberg, Darya Ståhl, Åsa Wiman, Filip Ventorp, Sophie Erhardt, Daniel Lindqvist

**Affiliations:** a Unit for Biological and Precision Psychiatry, Department of Clinical Sciences Lund, Lund University, Lund, Sweden; b Department of Psychiatry, Skåne University Hospital, Lund, Sweden; c Department of Gastroenterology and Nutrition, Department of Clinical Sciences, Skåne University Hospital, Malmö, Sweden; d Department of Psychiatry, Skåne University Hospital, Malmö, Sweden; e Department of Physiology and Pharmacology, Karolinska Institutet, Stockholm, Sweden

**Keywords:** Major depressive disorder, N-3 PUFAs, Probiotics, Nutraceuticals, Tryptophan, Kynurenine pathway

## Abstract

Activation of the kynurenine pathway (KP) with resulting accumulation of neuroactive metabolites may be a downstream pathophysiological mechanism of inflammatory depression. The aims of this study were to investigate KP metabolites in inflammatory depression compared to non-inflammatory depression and healthy controls, and whether these metabolites change with nutraceutical interventions with potential KP-modulating effects. We combined data from two antidepressant clinical trials: one with omega-3 polyunsaturated fatty acids (n-3 PUFAs) and one with a *Limosilactobacillus reuteri* probiotic supplement as the active intervention. Patients with Major Depressive Disorder (MDD) (n = 170) were stratified at baseline according to high-sensitivity C-reactive protein (hs-CRP) levels into an inflammatory (hs-CRP ≥1 mg/L, n = 127) and a non-inflammatory (hs-CRP <1 mg/L, n = 43) group. We also included 80 non-depressed healthy controls (HC). We investigated between-group differences in KP metabolites at baseline, treatment-associated changes in these biomarkers and how they relate to clinical response. At baseline, the inflammatory depression group had significantly elevated levels of quinolinic acid (QUIN) (p < 0.01), 3-hydroxykynurenine (3-HK) (p < 0.05), and kynurenine (p < 0.05) levels compared to both non-inflammatory depression and HCs. N-3 PUFAs, but not probiotics or placebo, significantly decreased QUIN (p < 0.05) and 3-HK (p < 0.05). Higher baseline levels and a larger treatment-associated decrease of several KP metabolites were associated with a better clinical response to n-3 PUFAs (p < 0.05). In contrast, probiotic supplementation was not associated with significant changes in KP metabolites, and biomarker associations with treatment response were limited in this cohort. We found evidence of KP activation in MDD, but only in an inflammatory subgroup, suggesting that these biological alterations may be specific to a subset of patients with low-grade inflammation. These findings encourage further investigations into whether, and how, biomarkers of KP activation may predict antidepressant response.

## Highlights

• MDD patients with low-grade inflammation had elevated kynurenine pathway metabolites.
• N-3 PUFA supplementation reduced quinolinic acid, kynurenic acid and 3-hydroxykynurenine.
• Baseline activation of the kynurenine pathway was associated with n-3 PUFA treatment response.
• Decrease in kynurenine pathway metabolites was associated with n-3 PUFA treatment response.

## 1 Introduction

Major Depressive Disorder (MDD) is a common cause of disability (Hall et al., 2024; Marwaha et al., 2023) with a lifetime prevalence of approximately 20% (Rush et al., 2006). Up to 50% of patients do not respond to first-line antidepressant treatment (Hall et al., 2024; Marwaha et al., 2023), and about 1/3 do not remit even after multiple treatment attempts (Rush et al., 2006). The slow development of new and targeted antidepressants may partly be due to the heterogeneity of MDD in terms of symptom presentation and pathophysiological mechanisms. In search for subtypes of MDD that may respond differently to targeted treatments, “inflammatory depression” has emerged as a possible candidate. This depression subtype is characterized by systemic low-grade inflammation, usually defined through modest high-sensitivity C-reactive (hs-CRP) elevations, metabolic disturbances, worse response to conventional antidepressants, and a symptom profile of fatigue, motivational deficits, sleep and appetite disturbances (Suneson et al., 2021). Previous studies have used hs-CRP cut-offs of either ≥1 mg/L (Jha et al., 2017; Nettis et al., 2021; Pan et al., 2022; Soderberg Veiback et al., 2025) or >3 mg/L (Felger et al., 2020; Rapaport et al., 2016; Suneson et al., 2023) to define low-grade inflammation in depression.

**Fig. 1:**
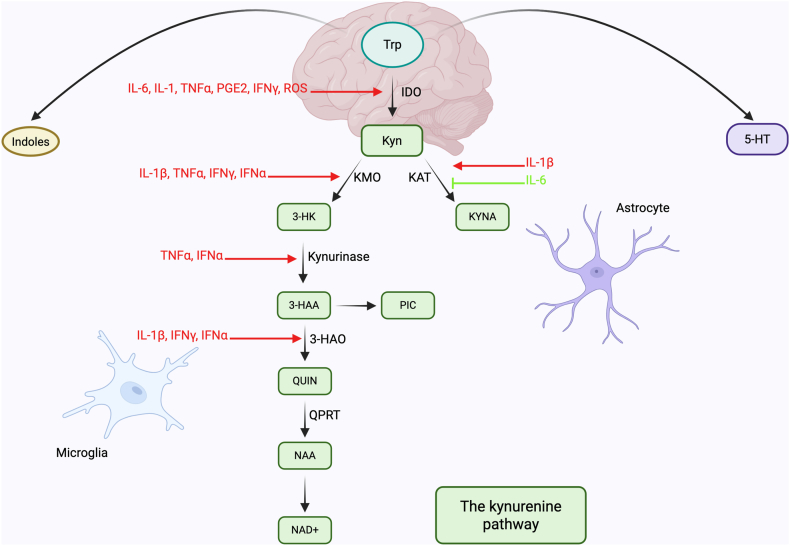
Tryptophan metabolism and the kynurenine pathway in inflammatory depression. Abbreviations: IDO, indolamine 2,3-dioxygenase; IFN, interferon; IL, interleukin; KYNA, kynurenic acid; KAT, kynurenine aminotransferase; KMO, kynurenine 3-monooxygenase; Kyn, kynurenine; NAA, nicotinamide; NAD+, nicotinamide adenine dinucleotide; PIC, picolinic acid; PGE2, prostaglandin E2; QUIN, quinolinic acid; QPRT, quinolinate phosphoribosyltransferase; ROS, radical oxygen species; TNF, tumour necrosis factor; Trp, tryptophan; 3-HAA, 3-hydroxyanthranilic acid; 3-HAO, 3-hydroxyanthranillate-3,4-dixogygenase; 3-HK, 3-hydroxykynurenine; 5-HT, serotonin.

Meta-analyses have demonstrated antidepressant effects of omega-3 polyunsaturated fatty acids (n-3 PUFAs) (Liao et al., 2019; Lu et al., 2024) that are potentially mediated via anti-inflammatory mechanisms (Mac Giollabhui et al., 2023; Mischoulon et al., 2022; Rapaport et al., 2016; Suneson et al., 2024). N-3 PUFAs reduce inflammation through several mechanisms, including the inhibition of pro-inflammatory cytokines, reducing pro-inflammatory eicosanoid synthesis, and producing anti-inflammatory and neuroprotective resolvins (Calder, 2013). Another promising antidepressant intervention is probiotic supplementation. A meta-analysis by Nikolova et al. indicated that probiotics are effective when used as an adjunctive, but not as a standalone, treatment (Nikolova et al., 2021). Although the antidepressant mechanisms of probiotics are unclear, they may also involve anti-inflammatory actions (Suneson et al., 2021). Despite promising clinical data in support of an antidepressant effect of n-3 PUFAs and probiotics, downstream mechanisms of these interventions are not fully understood. Moreover, biological markers predicting clinical response have not yet been validated. Some studies suggest that both n-3 PUFAs (Morgese et al., 2020; Tomczyk et al., 2024) and probiotics (Kazemi et al., 2019; Purton et al., 2021) affect the kynurenine pathway (KP) of tryptophan (Trp) metabolism which has been previously implicated in MDD pathophysiology (Correia and Vale, 2022; Hall et al., 2024; Savitz, 2020; Suneson et al., 2021). Trp can be metabolized in three principal ways: to serotonin; to anti-inflammatory signaling molecules indoles; and to kynurenine (Kyn) along the KP (Agus et al., 2018). The KP is involved in several important biological functions, including synthesis of immune-regulatory metabolites and the generation of cell energy (Agus et al., 2018; Correia and Vale, 2022; Moffett et al., 2020; Savitz, 2020). In the brain, the KP regulates neuroinflammation and neurotransmission – biological processes thought to be involved in MDD pathophysiology (Correia and Vale, 2022; Hall et al., 2024; Lucido et al., 2021; Savitz, 2020; Suneson et al., 2021). Pro-inflammatory cytokines may shift Trp metabolism in favor of the KP (Hall et al., 2024) through activation of indoleamine 2,3-dioxygenase (IDO) - an enzyme that converts Trp to Kyn (Suneson et al., 2021) (Fig. 1).

Previous studies measuring KP metabolites in MDD have generally included relatively small samples and reported inconsistent findings with either high (Erhardt et al., 2013; Nikkheslat et al., 2015; Sublette et al., 2011), low (Akkasheh et al., 2016; Ashok et al., 2017; Baker et al., 2018; Cathomas et al., 2021; Clark et al., 2016; Hennings et al., 2013; D. Krause et al., 2017; D. L. Krause et al., 2012; Meier et al., 2016; Ogyu et al., 2018; Savitz et al., 2015a,b,c), or unchanged (Dahl et al., 2015; Meier et al., 2016) levels in depressed patients compared to controls. Some of these discrepancies might be attributed to whether KP metabolites are measured peripherally or centrally, but it is also possible that they may be explained by the biological heterogeneity of MDD, i.e. that only a subgroup of MDD would display KP activation. In this study, we hypothesized that KP activation is more pronounced in a subset of depression with low-grade inflammation. We also investigated the relationship between KP metabolites and clinical response to n-3 PUFAs and probiotics.

## 2 Methods

### 2.1 Overview

This study included data from two previously published antidepressant trials conducted in Lund, Sweden, investigating the antidepressant effect of n-3 PUFAs (Lindahl et al., 2025; Suneson et al., 2024) and a *Limosilactobacillus reuteri* (*L*. *reuteri* DSM17938 and *L*. *reuteri* ATCC PTA 6475) probiotic supplement (Soderberg Veiback et al., 2025). In the former study (Suneson et al., 2024) all patients received add-on n-3 PUFAs and in the latter study (Soderberg Veiback et al., 2025) patients were randomized to either add-on *L*. *reuteri* or placebo. Both studies were pre-registered at clinicaltrials.gov (ref#NCT03143075 and ref#NCT03660280), and the study protocol for the n-3 PUFA study was pre-published (Suneson et al., 2022). Both studies were approved by the Ethical Review Board in Lund, Sweden (ref#2017/150 and ref#2018/379). The trials were externally monitored by Clinical Studies Sweden, Forum South, to ensure they met the ethical and scientific quality standards of the *Good Clinical Practice* guidelines.

### 2.2 Study design and participants

Detailed methodologies of the respective studies have been published elsewhere (Lindahl et al., 2025; Soderberg Veiback et al., 2025; Suneson et al., 2022, 2024). In summary, MDD patients were recruited between 2017 and 2023 by social media ads, radio, newspapers, and clinical referrals. A diagnosis of MDD was determined according to the DSM criteria by a physician-led diagnostic interview. Non-depressed healthy controls (HCs) were recruited between 2018 and 2022 through newspaper and social media ads. Blood samples were drawn at baseline and, in a subset of MDD patients, after 8 weeks of intervention. Also, a subset of the HCs had repeated blood sampling 8 weeks after baseline. The number of participants with repeated blood sampling (baseline and week 8) was 166, comprising 89 n-3 PUFA-treated patients, 33 probiotics-treated patients, 29 placebo-treated patients, and 15 HCs.

In the n-3 PUFA study, all patients received daily supplementation with capsules containing 2.2 g EPA, 400 mg DHA, 800 mg of other fatty acids and 10–24 mg tocopherol-rich extracts for 8 weeks alongside stable, ongoing pharmacological antidepressant treatment (Suneson et al., 2024). The main hypothesis of this study, which was partly confirmed (Suneson et al., 2024), was that patients with elevated (≥1 mg/L) hs-CRP would respond better to n-3 PUFAs than patients with low (<1 mg/L) hs-CRP. Patients with both high and low hs-CRP levels were eligible for this study. We utilized a match-mismatch design, stratifying patients in advance based on their baseline hs-CRP levels.

In the probiotics study, MDD subjects with a body mass index (BMI) ≥ 25 kg/m2 and baseline blood hs-CRP ≥1 mg/L were enrolled. Patients were randomized to receive active intervention with *L*. *reuteri* probiotic or placebo daily for 8 weeks alongside stable, ongoing antidepressant treatment (pharmacological treatment and/or cognitive behavioral therapy). In the main analysis, we did not find that *L*. *reuteri* was superior to placebo (Soderberg Veiback et al., 2025).

Full eligibility criteria for the n-3 PUFA and the probiotics study are shown in Supplementary Table 1 and 2, respectively.

Primary outcome measures in the n-3 PUFA study were baseline to week 8 change in total scores of the Hamilton Depression Rating Scale 17-item (HDRS-17) (Hamilton, 1960). In the probiotics study, co-primary outcome measures were change from baseline to week 8 in Montgomery-Åsberg Depression Rating Scale (MADRS-M) total scores (Montgomery and Asberg, 1979) and change in a composite score comprising three items from the Patient Health Questionnaire-9 (PHQ-9) (Kroenke et al., 2001) which have been associated with inflammatory depression (Frank et al., 2021; Fried et al., 2020a; Jokela et al., 2016; White et al., 2017) (items #3 sleep problems, #4 lack of energy, #5 appetite disturbances). The secondary outcome measures that were available for both studies were the Fatigue Severity Scale (FSS) (Krupp et al., 1989), the Insomnia Severity Index (ISI) (Bastien et al., 2001), the Generalized Anxiety Disorder 7-item scale (GAD-7) (Spitzer et al., 2006), and the PHQ-9 including the inflammatory composite score.

We used rating scales that were available from both studies (FSS, ISI, GAD-7, PHQ-9) when we analyzed the datasets combined (e.g. correlations between biomarkers and symptoms). When datasets were analyzed separately (e.g. associations between biomarkers and the effect of a specific intervention), we primarily analyzed the primary outcome measure of the respective study but also explored correlations between biomarkers and the secondary outcomes.

Treatment response was defined as ≥ 50% improvement on the MADRS-M (probiotics study) and the HDRS-17 (n-3 PUFA study) respectively. Since anhedonia symptoms may be associated with low-grade inflammation (Bekhbat et al., 2022; Lucido et al., 2021; Treadway, 2025), we also defined response as a ≥50% improvement on the Snaith-Hamilton Pleasure Scale (Snaith et al., 1995) (SHAPS), which was available only in the n-3 PUFA study. For this purpose, we applied the original binary scoring system in which ‘agree’ responses are scored as 0 and ‘disagree’ responses as 1, resulting in a total score ranging from 0 to 14, with higher scores indicating more severe anhedonia.

### 2.3 Blood sampling and biomarker analysis

EDTA blood samples were collected in the morning after an overnight fast and stored at −80 °C before being sent for analysis at the Department of Physiology and Pharmacology at Karolinska Institute, Stockholm, Sweden. A detailed description of the blood sampling procedures has been published elsewhere (Suneson et al., 2022). Plasma samples were thawed prior to UPLC-MS/MS analysis. Once thawed, 30 μl of the sample and 30 μl of internal standard (0.5 μM, or 5 μM for Trp) were mixed in 3.2% ammonia solution by vortexing for 15 s. Thereafter, 60 μl of 200 nM ZnSO_4_ (+4 °C) was added and mixed for 15 s, followed by the addition of 30 μl of methanol (+4 °C, UPLC grade), which was also mixed for 15 s. The resulting mixture was centrifuged for 10 min at 2300×*g* (+4 °C). Then, 30 μl of the resulting supernatant was mixed with 30 μl of 5% formic acid in water in Certified ClearGlass 12 × 32 mm vials (186005662CV, Waters).

Concentrations (μM) of the metabolites Trp, Kyn, kynurenic acid (KYNA), quinolinic acid (QUIN), picolinic acid (PIC), 3-hydroxykynurenine (3-HK), and nicotinamide (NAA) were measured using ultra-performance liquid chromatography–tandem mass spectrometry (UPLC-MS/MS). The system used was a Xevo TQ-XS triple quadrupole mass spectrometer (Waters, Manchester, UK), including a Z-spray electrospray interface and a Waters Acquity UPLC I-Class FTN system (Waters, MA, USA). The UPLC system was equipped with an Acquity UPLC HSS T3 column, dimensions 2.1 × 150 mm, 1.8 μm (part number: 186003540, Waters), and was set to a temperature of 50 °C. It was also equipped with a guard column (part number: 186003976, Waters) and an isolator column (part number: 186004476, Waters). Detailed descriptions of the analysis conditions, and the preparation of materials used for quality control samples, standards, and internal standards of metabolites, are reported elsewhere (Trepci et al., 2020).

Samples were analyzed using two mobile phases: the first containing 0.6% formic acid in water, and the second containing 0.6% formic acid in methanol. The flow rate was set to 0.3 ml/min, and the run time for each sample was 13.0 min. The autosampler was set to 5 °C, and 1.5 μl of each sample was injected into the UPLC-MS/MS system. The mass spectrometer operated in electrospray-positive multiple reaction monitoring (MRM) mode with a source temperature of 150 °C, capillary voltage of 0.4 kV, desolvation temperature of 600 °C, desolvation gas flow rate of 900 L/h, and detector gain of 1. Data processing was performed using MassLynx 4.2 software. Five re-runs were performed due to problems with the internal standard signal in the MS detector (increased intensity was observed during these runs). As a result, 42.8% of the samples were freeze-thawed once more before reanalysis. This included 58.8% of the healthy control samples, 48.5% of the n-3 PUFA baseline samples, 26.1% of the n-3 PUFA week 8 samples, 47.9% of the probiotics baseline samples, and 30.6% of the probiotics week 8 samples. The proportion of samples that underwent additional freeze–thaw cycles did not differ significantly between groups (chi-square test, p > 0.29), indicating that this factor is unlikely to have introduced systematic bias. Metabolite recordings from twenty biological samples were deemed unreliable post-analysis due to blood contamination, values outside the calibration curve, or large deviations in the internal standard. This issue most frequently concerned PA, for which values were often below the calibration curve. These measurements were excluded from the study. HC blood samples were analyzed two times; therefore, mean concentrations of each biomarker were calculated for this group.

Hs-CRP was measured using automated particle-based immunoassay as described elsewhere (Soderberg Veiback et al., 2025; Suneson et al., 2024).

### 2.4 Statistical analyses

All analyses were performed using the Statistical Package for the Social Sciences (SPSS), IBM Corp, released 2021 (IBM SPSS Statistics for Windows, Version 28.0, Armonk, NY: IBM Corp). We used natural logarithm (ln) transformation on non-normally distributed data when using parametric tests. Delta values of metabolites were calculated by subtracting the baseline from week 8 levels. To investigate associations between treatment response and biomarker levels we used non-parametric tests. Cross-sectional group-wise comparisons were done using one-way analysis of variance (ANOVA). Biomarkers were compared between inflammatory depression, non-inflammatory depression, and HCs, using a Bonferroni adjusted p-value of significance <0.02 (0.05/3). For all other analyses, the significance level was set to p < 0.05. Independent samples t-tests were used to compare baseline mean metabolite levels between responders and non-responders. These comparisons were conducted separately for the probiotics and n-3 PUFA groups. Effect sizes were assessed using Cohen's d. Changes in metabolite levels from baseline to week 8 were assessed using paired samples t-tests on ln-transformed metabolite values separately for each intervention group: (i) active probiotics treatment, (ii) n-3 PUFA treatment, and (iii) no active intervention (placebo and HCs combined). This allowed us to examine biomarker changes before and after each respective intervention and compare to change with no intervention.

To account for multiple testing in correlation analyses betwen biomarkers and symptoms and when comparing biomarkers between responders and non-responders, false discovery rate (FDR) correction was applied using the Benjamini–Hochberg procedure. Analyses were grouped into families of related tests, and FDR adjustment was performed within each family. In most cases, a family consisted of seven p-values (correlations between a symptom scale and the seven tryptophan biomarkers), whereas comparisons between responders and non-responders comprised fourteen p-values corresponding to the seven biomarkers assessed across two groups. FDR-adjusted p-values are provided in Supplementary Table 7.

## 3 Results

### 3.1 Demographic characteristics

**Fig. 2:**
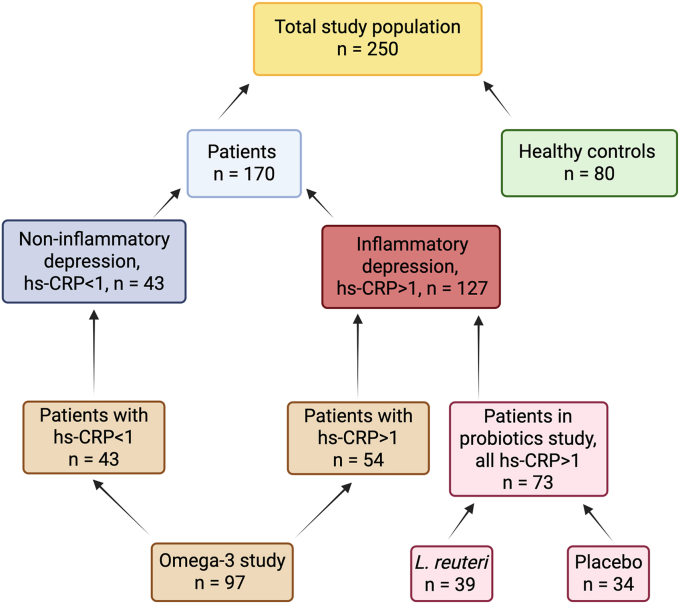
**Total study population**. This included subjects from the omega-3 and probiotics study. Subjects were eligible for inclusion in this study if they had available KP metabolites data at baseline. Abbreviations: hs-CRP, high sensitivity C-reactive protein; *L. reuteri*, *Lactobacillus reuteri*.

At baseline, biomarker data were available for 250 subjects, including 127 with inflammatory depression, 43 with non-inflammatory depression, and 80 HCs (Fig. 2). Patients with CRP ≥1 mg/L were classified into the inflammation group and those with CRP <1 mg/L into the non-inflammation group. Previous studies have used hs-CRP cut-offs of >1 mg/L to define inflammatory depression (Jha et al., 2017; Nettis et al., 2021; Pan et al., 2022). Ninety-seven patients received treatment with n-3 PUFAs, of whom 54 were in the inflammatory and 43 in the non-inflammatory depression group. Thirty-nine patients received *L*. *reuteri* probiotic, and 34 received placebo — all of these patients were included in the inflammatory group since this was an inclusion criterion for that study. In the non-inflammatory group, there were 9.3% HDRS-17 responders (all had received n-3 PUFAs). In the inflammatory group there were 37.0% HDRS-17 responders among those who received n-3 PUFAs and 17.9% MADRS responders among those who received probiotics.

For the group-wise comparisons, we conducted sensitivity analyses using an hs-CRP threshold of ≥3 mg/L to define inflammatory depression, yielding 64 patients in the inflammation group (CRP ≥3 mg/L) and 106 in the non-inflammation group (CRP <3 mg/L). With this cut-off, inflammatory depression still showed robustly elevated levels of Quin and 3-HK, but for some of the other biomarkers, group differences were attenuated or lost statistical significance. ANOVA tests with post-hoc Bonferroni correction are presented in Supplementary Table 6.

As expected, BMI differed significantly between the inflammatory depression group, the non-inflammatory depression group, and HCs (p < 0.001), with the highest mean BMI observed in the inflammatory depression group. Age did not differ significantly between groups (p = 0.30), nor did gender distribution (p = 0.20), with all three groups including ≥75% female subjects (Supplementary Table 3).

### 3.2 Baseline biomarker differences between inflammatory, non-inflammatory groups and healthy controls

**Fig. 3:**
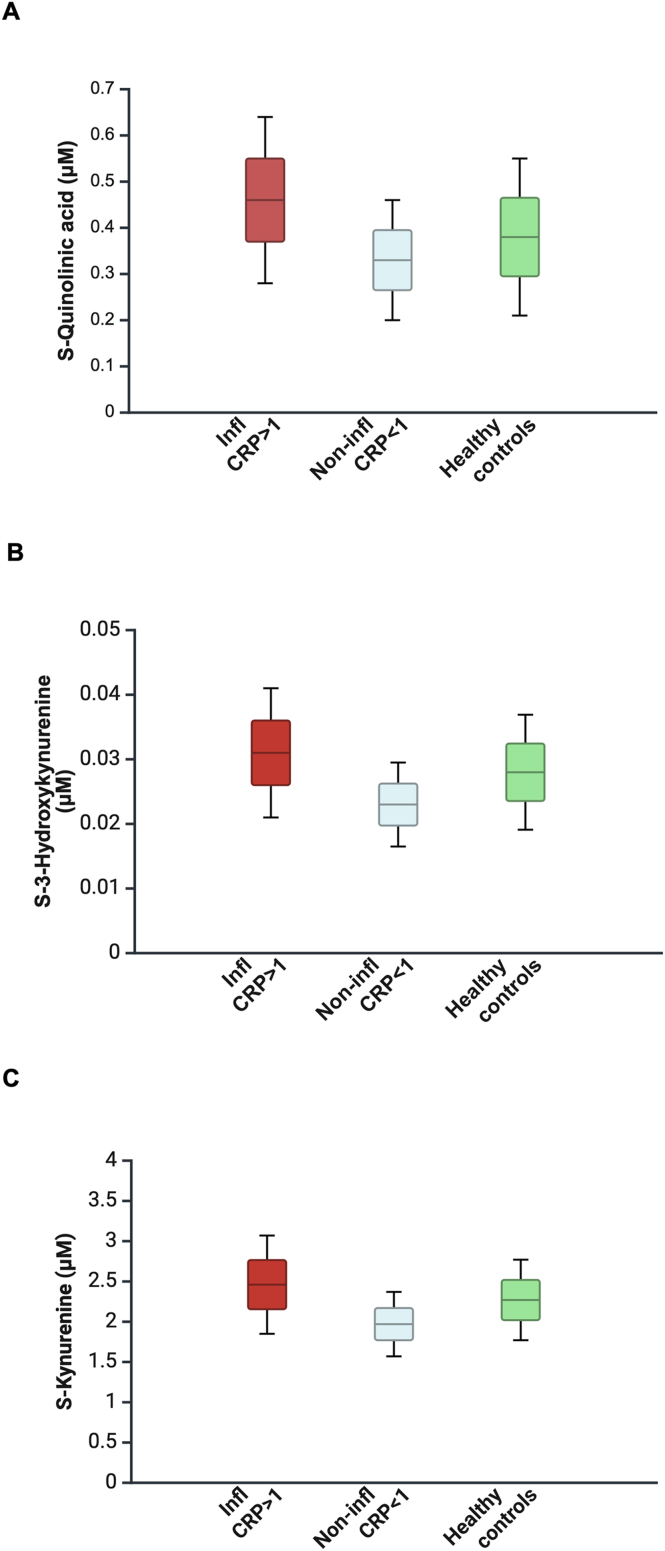
**Biomarkers significantly increased at baseline in inflammatory depression compared to non-inflammatory depression and healthy controls.** Biomarkers quinolinic acid (QUIN) (A), 3-hydroxikynurenine (3-HK) (B) and kynurenine (Kyn) (C) were significantly increased at baseline in inflammatory depression (hs-CRP ≥1) compared non-inflammatory depression (Hs-CRP<1) and healthy controls (all p < 0.05, Bonferroni adjusted). Boxplot graphs: boxes include mean absolute value (solid line) of biomarker at baseline and standard deviation from the absolute mean value indicated by the whiskers. Abbreviations: Infl CRP>1, inflammatory depression group; Non-infl CRP<1, non-inflammatory depression group.

Baseline levels of KP metabolites in the inflammatory depression group, non-inflammatory depression group, and HCs are shown in Table 1. The inflammatory depression group had significantly higher baseline levels of QUIN, 3-HK, and Kyn compared to both HCs (all p < 0.05) and the non-inflammatory depression group (all p < 0.001) (Fig. 3A–C). The non-inflammatory depression group had significantly lower KYNA levels compared to both HCs (p = 0.016) and the inflammatory depression group (p = 0.021). Trp levels were significantly lower in the non-inflammatory depression group compared to HCs (p = 0.041). No significant between-group differences were found for NAA and PIC (all p > 0.080).

**Table 1:** **Group-wise comparison of baseline biomarkers between inflammatory depression, non-inflammatory depression and healthy controls.** Mean absolute concentrations of biomarkers measured at baseline. Subjects stratified per baseline blood high-sensitivity C-reactive protein (hs-CRP) into inflammatory depression (hs-CRP≥1 mg/L), non-inflammatory depression (hs-CRP<1 mg/L) and healthy controls. Group-wise comparisons conducted with analysis of variance (ANOVA) tests. *Post hoc* Bonferroni corrected the level of statistical significance of the ANOVA tests.

Mean (SD) of biomarker concentrations at baseline			
Variable	Inflammatory depression	Non-inflammatory depression	Healthy controls
**Evaluated subjects**, *N*	**127**	**43**	**80**
**NAA** (μM)	**0.42** (0.19)	**0.41** (0.12)	**0.37** (0.15)
**PIC** (μM)	**0.015** (0.0010)	**0.015** (0.0089)	**0.016** (0.0079)
**QUIN** (μM)	**0.46** (0.18)	**0.33** (0.13)	**0.38** (0.17)
**KA** (μM)	**0.057** (0.020)	**0.048** (0.021)	**0.059** (0.022)
**Trp** (μM)	**43.50** (7.94)	**41.06** (5.58)	**44.35** (6.10)
**Kyn** (μM)	**2.46** (0.61)	**1.97** (0.40)	**2.27** (0.50)
**3-HK** (μM)	**0.031** (0.0103)	**0.023** (0.0065)	**0.028** (0.0089)

*Post hoc* Bonferroni adjusted p-values are reported			
Biomarker	Groups (A)	Groups (B)	p-value
**NAA**	Healthy controls	Non-inflammatory depression	0.52
		Inflammatory depression	0.082
	Non-inflammatory depression	Healthy controls	0.52
		Inflammatory depression	1.00
	Inflammatory depression	Healthy controls	0.082
		Non-inflammatory depression	1.00
**PIC**	Healthy controls	Non-inflammatory depression	1.00
		Inflammatory depression	1.00
	Non-inflammatory depression	Healthy controls	1.00
		Inflammatory depression	1.00
	Inflammatory depression	Healthy controls	1.00
		Non-inflammatory depression	1.00
**QUIN**	Healthy controls	Non-inflammatory depression	0.47
		Inflammatory depression	**0.002**
	Non-inflammatory depression	Healthy controls	0.47
		Inflammatory depression	**<0.001**
	Inflammatory depression	Healthy controls	**0.002**
		Non-inflammatory depression	**<0.001**
**KA**	Healthy controls	Non-inflammatory depression	**0.016**
		Inflammatory depression	1.00
	Non-inflammatory depression	Healthy controls	**0.016**
		Inflammatory depression	**0.021**
	Inflammatory depression	Healthy controls	1.00
		Non-inflammatory depression	**0.021**
**Trp**	Healthy controls	Non-inflammatory depression	**0.041**
		Inflammatory depression	1.00
	Non-inflammatory depression	Healthy controls	**0.041**
		Inflammatory depression	0.15
	Inflammatory depression	Healthy controls	1.00
		Non-inflammatory depression	0.15
**Kyn**	Healthy controls	Non-inflammatory depression	**0.010**
		Inflammatory depression	**0.048**
	Non-inflammatory depression	Healthy controls	**0.010**
		Inflammatory depression	**<0.001**
	Inflammatory depression	Healthy controls	**0.048**
		Non-inflammatory depression	**<0.001**
**3-HK**	Healthy controls	Non-inflammatory depression	**0.013**
		Inflammatory depression	**0.027**
	Non-inflammatory depression	Healthy controls	**0.013**
		Inflammatory depression	**<0.001**
	Inflammatory depression	Healthy controls	**0.027**
		Non-inflammatory depression	**<0.001**

Missing data: NAA (n = 1), PA (n = 6), QA (n = 2), KA (n = 2), Trp (n = 2), Kyn (n = 3). Abbreviations: BMI, body-mass index; Infl, inflammation; KA, kynurenic acid; Kyn, kynurenine; NAA, nicotinamide; Non-infl, non-inflammation; PA, picolinic acid; QA, quinolinic acid; s.d., standard deviation; Trp, tryptophan; 3-HK, 3-hydroxykynurenine.

### 3.3 Correlations between baseline biomarkers and symptom severity

In the cohorts combined, we found no significant correlations at baseline between any of the biomarkers and symptom severity using the rating scales that were available for both study cohorts (FSS, ISI, GAD-7, PHQ-9, and Inflammatory Depression Scale) (all p > 0.082).

We found a significant negative correlation between baseline SHAPS score and baseline Trp (rho = −0.27, p = 0.007, n = 95). SHAPS was available only in the n-3 PUFA cohort.

### 3.4 Correlations between baseline biomarkers and treatment response

In Sections 3.4, 3.5, treatment response is analyzed. These sections are therefore structured according to the specific cohort being examined, that is, they are subdivided based on the intervention received by the included participants. Several p-values from correlation analyses of treatment response did not remain significant after FDR adjustment; however, given the exploratory nature of these analyses, unadjusted p-values are reported in the main manuscript. However, Benjamini–Hochberg FDR-adjustment of significant correlations are provided in Supplementary Table 7.

#### 3.4.1 N-3 PUFA study

Treatment-associated change in HDRS-17 score was significantly correlated with baseline KYNA (rho = −0.22, p = 0.042, n = 88), Kyn (rho = −0.22, p = 0.043, n = 88), and 3-HK (rho = −0.21, p = 0.049, n = 90), indicating that higher baseline levels of these biomarkers were associated with greater improvement in depressive symptoms. Responders (≥50% reduction in HDRS-17 total score from baseline to week 8) to n-3 PUFAs (n = 24) had significantly higher baseline levels of QUIN (p = 0.038), KYNA (p = 0.003), Kyn (p = 0.003), and 3-HK (p = 0.025) compared to non-responders (n = 64 for QUIN, KYNA, and Kyn; n = 66 for 3-HK). There were no significant correlations between baseline biomarkers and change in SHAPS (all p > 0.096).

There was a significant negative correlation between baseline QUIN and change in FSS (rho = −0.24, p = 0.023, n = 88) and change in ISI (rho = −0.23, p = 0.033, n = 88). Baseline KYNA also correlated significantly with change in FSS (rho = −0.32, p = 0.003, n = 88), GAD-7 (rho = −0.21, p = 0.049, n = 88), and ISI (rho = −0.21, p = 0.047, n = 88). Baseline Kyn correlated significantly with change in FSS (rho = −0.22, p = 0.039, n = 88) and ISI (rho = −0.29, p = 0.005, n = 88). Baseline 3-HK correlated significantly with change in FSS (rho = −0.27, p = 0.012, n = 90) and ISI (rho = −0.21, p = 0.042, n = 90). All these correlations between baseline biomarkers and treatment-associated change in symptoms were negative, indicating that higher baseline levels of these biomarkers were associated with greater symptom improvement following n-3 PUFA treatment. All other correlations between baseline biomarkers and change in symptoms were non-significant (data not shown).

#### 3.4.2 Probiotics study

In the probiotics group, which included seven responders (≥50% reduction in MADRS-M total score from baseline to week 8) and 32 non-responders, there were no significant correlations between baseline biomarkers and change in MADRS score (all p > 0.15; n = 37 for all biomarkers except PIC, for which n = 36), and there were no significant differences in baseline levels in any of the biomarkers between responders and non-responders (all p > 0.12). There was a significant positive correlation between baseline QUIN and change in inflammatory depression symptoms (total composite score of three items from PHQ-9 defined in section 2.2) (rho = 0.40, p = 0.018, n = 34) and total PHQ-9 score (rho = 0.38, p = 0.027, n = 34). Baseline Kyn also correlated significantly with change in PHQ-9 (rho = 0.37, p = 0.032, n = 34). A positive correlation here indicates that lower baseline levels of these biomarkers were associated with greater symptom improvement. All other correlations between baseline biomarkers and change in symptoms were non-significant (data shown).

### 3.5 Correlations between change in biomarkers and change in symptoms

#### 3.5.1 N-3 PUFA study

In all patients who received n-3 PUFAs, QUIN, KYNA, and 3-HK all decreased significantly with treatment (all p < 0.05, Table 2). There were no significant correlations between changes in any of the biomarkers and change in HDRS-17 scores (all p > 0.070). There was a negative correlation between change in Trp and change in anhedonia symptoms (rho = −0.218, p = 0.043, n = 86). Moreover, QUIN (p = 0.010, n = 44) and 3-HK (p = 0.003, n = 46) decreased significantly from baseline to week 8 among SHAPS responders (n = 46) (response defined as ≥50% reduction in total score from baseline to week 8). These biomarkers did not significantly change with treatment in SHAPS non-responders (all p > 0.61). The treatment-associated decrease in QUIN in SHAPS responders was significantly greater compared to non-responders (Cohen's d = −0.44, p = 0.046). Change in FSS score correlated with both change in QUIN (rho = 0.24, p = 0.025, n = 86) and change in 3-HK (rho = 0.22, p = 0.040, n = 88), indicating that a decrease in these biomarkers was associated with improvements in symptoms of fatigue. All other correlations between change in biomarkers and change in symptoms were non-significant (data shown).

**Table 2:** **Change in biomarkers for patients (n = 89) receiving n-3 PUFAs.** Mean absolute biomarker concentrations measured at baseline (BL) and end of study (week 8). Paired samples *t*-test was performed using logarithmically transformed values.

Biomarker	Time	Mean absolute concentrations (μM) (s.d)	Paired samples *t*-test	
			Point estimate - *Cohen's d*	Two-sided p
**NAA**	BL	0.43 (0.17)	0.15	0.17
	w8	0.41 (0.16)		
**PIC**	BL	0.014 (0.0074)	0.086	0.43
	w8	0.014 (0.0073)		
**QUIN**	BL	0.38 (0.15)	0.26	**0.017∗**
	w8	0.34 (0.13)		
**KA**	BL	0.053 (0.020)	0.30	**0.006∗∗**
	w8	0.049 (0.020)		
**Trp**	BL	41.89 (6.03)	0.004	0.97
	w8	42.57 (7.30)		
**Kyn**	BL	2.17 (0.46)	0.20	0.060
	w8	2.11 (0.51)		
**3-HK**	BL	0.027 (0.0085)	0.26	**0.016∗**
	w8	0.025 (0.0083)		

∗p < 0.05; ∗∗p < 0.01; ∗∗∗p < 0.001.

All decimal values given to two significant figures. Missing data: NAA (n = 1), QUIN (n = 2), KA (n = 2), Trp (n = 2), LK (n = 2), PA (n = 5). Abbreviations: BL, baseline; KA, kynurenic acid; Kyn, kynurenine; NAA, nicotinamide; n-3 PUFAs, omega-3 polyunsaturated fatty acids; PIC, picolinic acid; QUIN, quinolinic acid; s.d., standard deviation; Trp, tryptophan; 3-HK, 3-hydroxykynurenine.

#### 3.5.2 Probiotics study

In all patients who received *L*. *reuteri*, there was a significant decrease in NAA (p < 0.05, Supplementary Table 4). Also, in those with no intervention (HCs with repeated sampling and MDD patients receiving placebo), there was a decrease in NAA (p < 0.01, Supplementary Table 5). No other biomarkers changed significantly with either *L*. *reuteri*, placebo, or in HCs with repeated blood samples. There was a significant negative correlation between change in QUIN and change in MADRS-M scores (rho = −0.38, p = 0.031), indicating that an increase in QUIN was associated with a decrease in depressive symptoms following probiotic supplementation. No other significant correlations between changes in biomarkers and changes in MADRS scores were observed in this group. There were no significant correlations between changes in biomarkers and changes in secondary outcome measures (FSS, ISI, GAD-7, PHQ-9) in the probiotics group (data not shown).

## 4 Discussion

In this study, we found evidence of KP activation in MDD – but only in patients with low-grade inflammation. N-3 PUFA treatment decreased several KP metabolites that were elevated in the inflammatory depression group before treatment. Treatment-associated changes in these biomarkers correlated with greater clinical response to n-3 PUFAs, particularly improvement in anhedonia and fatigue.

The various metabolites of the KP have distinct biological effects, in some cases depending on cell type. In immune cells, such as microglia and macrophages, metabolism of Kyn leads to an increase in pro-inflammatory metabolites 3-HK and QUIN. 3-HK promotes oxidative stress and QUIN, an agonist to the glutamatergic N-methyl-D-aspartate (NMDA) receptor, contributes to neurotoxicity and dysregulated glutamatergic neurotransmission (Agus et al., 2018; Correia and Vale, 2022; Ogyu et al., 2018; Suneson et al., 2021). Kyn metabolism in astrocytes increases synthesis of NMDA antagonist KYNA, which may exert neuroprotective effects and potentially attenuate the neurotoxic effects of QUIN (Correia and Vale, 2022; Liang et al., 2022; Suneson et al., 2021). However, KYNA has also been associated with cognitive dysfunction in other psychiatric conditions (Liang et al., 2022; Plitman et al., 2017). Previous studies suggest that KP metabolites are tightly linked to inflammation, and inflammation, in turn, may promote depressive symptoms (Savitz, 2020; Suneson et al., 2021). Despite this established link, previous studies have shown inconsistent results regarding the direction of KP metabolite changes in MDD, with reports of either elevated, decreased or unchanged levels compared to controls, as summarized in a recent meta-analysis (Ogyu et al., 2018). In the current study, we found significant increases in several KP metabolites in inflammatory depression compared to both non-inflammatory depression and HCs. Additionally, we observed lower Trp levels in non-inflammatory depression compared to controls, indicating a distinct biochemical phenotype that could be linked to reduced availability of the serotonin precursor rather than an overactivation of the KP. Previous studies have reported associations between inflammation and KP metabolites in patients with depression, with inflammation shifting Trp metabolism towards the KP through cytokine activation of IDO (Cho et al., 2017; Clark et al., 2016; Dahl et al., 2015; Erhardt et al., 2013; D. Krause et al., 2017; D. L. Krause et al., 2012; Meier et al., 2016; Nikkheslat et al., 2015; Quak et al., 2014; Savitz et al., 2015; Savitz et al., 2015; Savitz et al., 2015; Young et al., 2016). We found that 3-HK, Kyn and QUIN were all significantly increased in inflammatory depression compared to both controls and non-inflammatory depression. As for QUIN, there are some previous reports of unchanged (Dahl et al., 2015) or lower (Clark et al., 2016) levels in depression compared to controls (Cathomas et al., 2021; Dahl et al., 2015). Similar inconsistencies have been reported for Kyn (Cathomas et al., 2021; Hennings et al., 2013; H. Liu et al., 2018; Quak et al., 2014) and 3-HK (Cathomas et al., 2021; Young et al., 2016) levels. Our findings indicate that KP activation is not a universal feature of all MDD cases but appears to be more pronounced in a specific subtype characterized by low-grade inflammation. Previous inconsistencies across studies may stem from a lack of stratification of MDD patients based on baseline inflammation, overlooking depression heterogeneity in study designs (Lucido et al., 2021). We note, however, that some previous studies reported elevated QUIN levels in depression, regardless of inflammatory status, compared to controls (D. Krause et al., 2017; H. Liu et al., 2018; Meier et al., 2016; Rapaport et al., 2016; Savitz et al., 2015; Savitz et al., 2015; Savitz et al., 2015), challenging the idea that KP activation is limited to a subgroup with inflammatory depression. On the other hand, there are also several examples of a direct correlation between QUIN and inflammatory markers, suggestive of a dose-response relationship (Erhardt et al., 2013; Savitz et al., 2015; Savitz et al., 2015).

We found that QUIN, 3-HK and KYNA decreased following treatment with anti-inflammatory n-3 PUFAs, but not with *L*. *reuteri* probiotic or placebo. Elevated baseline QUIN and 3-HK, and a treatment-associated decrease in these biomarkers, was associated with a greater improvement in anhedonia, fatigue and insomnia following n-3 PUFA supplementation. These results indicate that KP metabolites may predict antidepressant treatment response to n-3 PUFAs, particularly improvement in symptoms associated with an inflammatory depression subtype – such as anhedonia, fatigue and sleep disturbances (Frank et al., 2021; Fried et al., 2020a; Jokela et al., 2016; White et al., 2017).

We did not conduct formal mediation analyses and therefore cannot draw definitive mechanistic conclusions about the biological underpinnings of the antidepressant response to n-3 PUFAs. We note, however, that N-3 PUFAs are known to have anti-inflammatory properties (Calder, 2013; Hutchinson et al., 2020; Suneson et al., 2021) that could lead to a reduction in KP activation (Calder, 2013). In animal studies, n-3 PUFAs reduce the production of pro-inflammatory IDO-activating substances – including the eicosanoid prostaglandin E2 (PGE2) (Calder, 2013; Müller and Schwarz, 2007) and cytokines IL-6, TNF-α and IL-1β (Calder, 2013; Hall et al., 2024) – which results in less KP activation since IDO initiates this pathway (Fig. 1). Morgese et al. showed that n-3 PUFAs are important for regulating inflammation, KP metabolite levels and symptoms of depression, as animals with dietary deficiency in n-3 PUFAs developed depressive-like behavior, systemic inflammation and increased levels of intracerebral KP metabolites compared to animals not deficient in n-3 PUFAs (Morgese et al., 2020). Altogether these results suggest that the antidepressant effects of n-3 PUFA may be mediated via anti-inflammatory mechanisms and reduced KP activation. However, since we did not conduct formal mediation analyses more studies are needed to confirm this hypothesis. The observed Spearman correlation coefficients were generally small to moderate in magnitude (ρ ≈ 0.20–0.32), indicating modest effect sizes that are typical for biomarker–symptom associations in heterogeneous clinical populations. While these effects are not large, they may still be clinically meaningful in the context of multifactorial depression biology and could contribute to multivariate biomarker models predicting treatment response.

We found that higher baseline levels of several KP metabolites were associated n-3 PUFA treatment-related improvements in sleep difficulties. Cho et al. (2017) found that sleep disturbances are associated with low-grade inflammation and increased KP activation, suggesting a link between KP activation and a depression subtype characterized by sleep disturbances (Cho et al., 2017). A recent review by Shimizu et al. showed that dietary supplementation with n-3 PUFAs improves sleep quality, particularly by reducing the amount of time spent awake and increasing the amount of time spent asleep during the night (Shimizu et al., 2024). Increased nighttime wakefulness can lead to daytime fatigue which is another symptom related to inflammatory depression (Suneson et al., 2021). In line with these results, our group previously showed that add-on n-3 PUFAs improve fatigue and insomnia in MDD (Suneson et al., 2024). We suggest that higher baseline KP metabolites indicate a better response to n-3 PUFAs on these specific symptoms, and if our results are replicated in independent cohorts these biomarkers could aid in tailoring n-3 PUFA antidepressant interventions according to biology and symptoms.

While there is some preclinical evidence suggesting an anti-inflammatory and KP-modulating effect of *L*. *reuteri* (Han et al., 2020; Jang et al., 2019; Lee et al., 2023; Schwarcz et al., 2024) our clinical results generally did not support this. In the current study, the effects of *L*. *reuteri* on KP metabolites were less robust and not consistent with the observed effects of n-3 PUFA treatment on KP activation. Reasons for this are unclear, but we note that preclinical results also differ with regards to the effects of *L*. *reuteri* on KP metabolites, with one study reporting increased KYNA synthesis by *L*. *reuteri* (Schwarcz et al., 2024) and another reporting decreased KYNA levels in the brain of mice expressing depressive-like behavior after treatment with *L*. *reuteri* (Lee et al., 2023). We found no effects on KYNA levels in subjects treated with *L*. *reuteri*. As reviewed by Liu et al., no *Limosilactobacillus* strains are inherently beneficial or harmful in the context of depression. Instead, their effects are influenced by the composition and balance of host's gut microbiome species (L. Liu et al., 2022). While we observed a significant reduction in NAA following probiotic intervention, the same pattern was observed in depressed patients receiving placebo and in HCs without any intervention. This suggests that the decrease in NAA may not be directly attributed to the effects of probiotic supplementation but could instead result from regression to the mean or natural variations. NAA is a byproduct of QUIN metabolism (Fig. 1) and plays a role in cellular energy reactions involving nicotinamide adenine dinucleotide (NAD+). NAD+ is one of the body's most prevalent metabolites, primarily due to its critical role in energy production, and as most NAD+ is derived from reactions recycling NAA rather than from KP activity (Moffett et al., 2020; Xie et al., 2020). Further research is advised to specify in what way probiotic supplementation and the gut microbiome may interact to alleviate symptoms of depression, and whether this is related to alterations in KP activity. Unlike the findings with n-3 PUFAs, lower baseline QUIN and Kyn were associated with greater improvements in the probiotic trial. Although these findings require further replication, they suggest that the two interventions may target different biological pathways. N-3 PUFAs appear to benefit patients with elevated inflammation and KP activity, while *L*. *reuteri* might be more effective for those without significant KP activation.

Patients with inflammatory depression in our sample also had higher BMI than both non-inflammatory MDD and healthy controls, which we interpret as part of an immuno-metabolic phenotype rather than a confounder (Capuron et al., 2017). Rather than “factoring out” BMI in our analyses, we interpret increased BMI as an inherent feature of inflammatory depression, as adiposity likely contributes to—and is closely intertwined with—alterations in inflammatory and KP biomarkers. Adipose tissue functions as an immunologically active organ, and given the design of the current study, we cannot fully disentangle the relative causal contributions of elevated BMI versus inflammation. When expanded and metabolically dysregulated as is often the case with obesity, adipose tissue secretes pro-inflammatory cytokines and adipokines promoting chronic low-grade inflammation, stimulating hepatic CRP production and inducing indoleamine 2,3-dioxygenase, thereby shifting tryptophan metabolism towards the kynurenine pathway (Coelho et al., 2013; Dantzer, O'Connor, Freund, Johnson and Kelley, 2008). Moreover, both increased adiposity and low-grade inflammation may arise downstream from metabolism and composition of gut microbiota (Geurts et al., 2014). When adjusting for BMI, the relationship between inflammation and depression have been shown to be attenuated (Fried et al., 2020b), but in a comprehensive systematic review (Osimo et al., 2019) BMI was not associated with low-grade inflammation in depression. We argue that statistical adjustment for BMI risks removing variance that is mechanistically related to the inflammatory depression construct we aimed to study.

Strengths of the study include a relatively large sample size and repeated biomarker measurements, which increased our ability to robustly assess the effects of nutraceuticals on KP metabolite levels and their associations with symptom changes during the interventions. However, a limitation is the extensive number of statistical tests conducted for exploratory purposes, which increased the risk of false positives due to multiplicity. To mitigate this, we applied a post hoc Bonferroni correction for baseline group comparisons and treatment response correlations adjusted for multiple comparisons (FDR) are provided in Supplementary Table 7. Nevertheless, these findings remain exploratory and require replication. Another potential limitation of the study is that all patients in the non-inflammatory MDD subgroup were recruited from the n-3 PUFA trial. However, we do not believe this introduced significant bias in the baseline group-wise comparisons. This is because the recruitment for both trials was conducted largely in parallel, targeting a similar population, with identical blood sampling and assay procedures across trials. Moreover, the probiotic study included a small number of responders, limiting statistical power; therefore, the absence of observed effects on the KP in the probiotics cohort should be considered inconclusive rather than evidence of no effect. During analysis, some blood samples had to be re-frozen and re-thawed. However, this should not have affected metabolite concentrations as a previous study examining the effects of repeated cycles of freeze-thawing on the stability of KP metabolites found that concentrations of Trp, Kyn, KA, 3-HK, PA, NAA remained stable, even after four cycles of freeze-thawing were repeated (Trepci et al., 2020). Finally, since we only analyzed KP metabolites in blood samples, we cannot extrapolate these findings to KP-related biological processes in the central nervous system.

## 5 Conclusions

In this study, we showed that the KP is activated in a subtype of inflammatory depression. We propose that specific KP metabolites could serve as biomarkers for this subtype and predict antidepressant response to n-3 PUFAs. Depressive symptoms linked to inflammatory depression improved alongside the normalization of KP metabolites following n-3 PUFA treatment. Our findings highlight the significance of defining depression subgroups, which could lead to the development of novel diagnostic tools and targeted treatments for patients who currently lack effective options, such as those with inflammatory depression.

## CRediT authorship contribution statement

**Jesper Lindahl:** Writing – original draft, Visualization, Investigation, Formal analysis, Data curation, Conceptualization. **Gustav Söderberg Veibäck:** Writing – review & editing, Investigation. **Klara Suneson:** Writing – review & editing, Investigation. **Wilma Blanking:** Writing – original draft, Visualization, Data curation. **Johanna Tjernberg:** Writing – review & editing, Project administration, Investigation. **Darya Ståhl:** Writing – review & editing, Project administration, Investigation, Data curation. **Åsa Wiman:** Writing – review & editing, Investigation. **Filip Ventorp:** Writing – review & editing, Supervision. **Sophie Erhardt:** Writing – review & editing, Supervision, Investigation, Funding acquisition. **Daniel Lindqvist:** Writing – original draft, Supervision, Methodology, Investigation, Funding acquisition, Formal analysis, Data curation, Conceptualization.

## AI declaration

During the preparation of this work the authors used the large language model ChatGPT (GPT-5, OpenAI, San Francisco, CA, USA) solely for grammar and spelling suggestions and to improve flow, readability and language. After using this tool, the authors reviewed and edited the content as needed and take full responsibility for the content of the publication.

## Funding

The study was funded by the 10.13039/501100004359Swedish Research Council (grant number 2020-01428), Swedish governmental funding of clinical research (10.13039/100001424ALF), the OM Persson Foundation, the Ellen and Henrik Sjöbring Foundation, the Söderström Königska Foundation, the Lions Research Foundation, the 10.13039/501100007687Swedish Society of Medicine, Skåne University Hospital Donations, 10.13039/501100013896BioGaia 10.13039/100024877AB, Dr P Håkansson's foundation**,** the Per-Eric and Ulla Schybergs Foundation, and the Professor Bror Gadelius memorial fund.

## Declaration of competing interest

The authors declare that they have no known competing financial interests or personal relationships that could have appeared to influence the work reported in this paper.

## Data Availability

Data cannot be made freely available, in accordance with the Swedish Public Access to Information and Secrecy Act, but requests of data can be sent to registrator@lu.se.
