# Supplementary material for: Kynurenine pathway metabolites are increased in inflammatory depression and decrease with omega-3 treatment
Source: Brain Behav Immun Health. 2026 Mar 25;53:101221. doi: 10.1016/j.bbih.2026.101221 (PMC13066792; doi:10.1016/j.bbih.2026.101221)
Supplement: Multimedia component 1 [file mmc1.docx]

Supplementary Tables to ”Kynurenine pathway metabolites are increased in inflammatory depression and decrease with omega-3 treatment”

Supplementary Table 1. **Eligibility criteria for the Omega-3 study**

| **Inclusion criteria** | **Exclusion criteria** |
| --- | --- |
| Age 18–80 years | Serious medical illness that could, in the investigator's opinion, jeopardise treatment response or interpretation of study results |
| Current unipolar depressive episode according to DSM-V criteria, symptom duration > 4 weeks | Allergy to the study intervention |
| HDRS-17 score ≥ 15 | Active infection |
| Clinical global impression severity score ≥ 3 | Pregnancy or breast-feeding |
| Stable antidepressant or mood stabilising treatment ≥ 6 weeks prior to participation | Diagnosed psychotic or bipolar disorder, dementia, cognitive disability, or individuals who lack the ability to make informed decisions due to other conditions |
| Patients agree to not significantly modify one's diet during the course of the study | Ongoing electroconvulsive therapy |
|  | Anticoagulant treatment or known bleeding disorder |
|  | Current, serious suicidal or homicidal risk according to the investigator’s judgment |
|  | Substance use disorder, except nicotine or caffeine, 3 months prior to screening visit |
|  | Any medications that could confound the biomarker analyses, within 1 week of baseline or throughout the trial, including: regular intake of NSAIDs or COX-2 inhibitors, any use of oral steroids, immunosuppressants, chemotherapy, interferon. Patients will be instructed against intake of NSAIDs (including Aspirin) or COX-2 inhibitors during the 24 h preceding a visit including biomarker assessment visit |
|  | Intake of n-3 fatty acid supplementation ≥ 3 consecutive days in the month prior to screening visit |
|  | Initiation of psychotherapy ≤ 4 weeks of start of study or plan to start psychotherapy during study |
|  | Participation in other clinical studies with ongoing study visits |

Abbreviations: COX-2, cyclooxygenase-2; DSM-V, Diagnostic and Statistical Manual of Mental Disorders edition V; HDRS-17, Hamilton Depression Rating Scale 17-score questionnaire; NSAIDs, nonsteroidal anti-inflammatory drugs.

Supplementary Table 2. **Eligibility criteria** **for the Probiotics study**

| **Inclusion critera** | **Exclusion criteria** |
| --- | --- |
| Age 18-85 | Serious medical illness that could, in the investigator's opinion, jeopardise treatment response or interpretation of study results |
| Depressive episode according to the DSM-V criteria, symptom duration > 4 weeks | Allergy to the study intervention |
| MADRS-M score ≥18 | Active infection |
| Clinical global impression severity score ≥ 3 | Pregnancy |
| Stable, ongoing antidepressant medication or in cognitive behavioural therapy for > 4 weeks | Diagnosed psychotic or bipolar disorder, personality disorder, dementia, cognitive disability, or individuals who lack the ability to make informed decisions due to other conditions |
| Patients agree to not significantly modify one's diet during the course of the study | Ongoing electroconvulsive therapy |
| A hs-CRP value ≥ 1mg/L and body mass index ≥ 25 kg/m^2^ | Current, serious suicidal risk according to investigator’s judgement |
|  | Diagnosed substance use disorder, except nicotine or caffeine, in the previous 3 months prior to screening visit |
|  | Any medications that could confound the biomarker analyses, within 1 week of baseline or throughout the trial, including: regular intake of NSAIDs or COX-2 inhibitors, any use of oral steroids, immunosuppressants, chemotherapy, interferon. Patients will be instructed against intake of NSAIDs (including Aspirin) or COX-2 inhibitors during the 24 h preceding a visit including biomarker assessment visit |
|  | Treatment with antibiotics ≤ 4 weeks prior to baseline visit |
|  | Ingested foods or supplementation with probiotics > 2 weeks before baseline visit |
|  | Active participation in other clinical studies with ongoing study visits |

Abbreviations: COX-2, cyclooxygenase-2; DSM-V, Diagnostic and Statistical Manual of Mental Disorders edition V; hs-CRP, high sensitivity C-reactive protein; MADRS-M, Montgomery-Åsberg Depression Rating Scale; NSAIDs, nonsteroidal anti-inflammatory drugs.

Supplementary Table 3. **Demographics.** Patients with available biological data at baseline in the omega-3 study and the probiotics study stratified per baseline blood high-sensitivity C-reactive protein (hs-CRP) into inflammatory depression (Infl CRP>1 mg/L), non-inflammatory depression (Non-infl CRP<1 mg/L) and healthy controls.

| **Variable** | **Infl CRP>1** | **Non-infl CRP<1** | **Healthy controls** | **p-value** |
| --- | --- | --- | --- | --- |
| **Evaluated subjects,** *N* | **127** | **43** | **80** |  |
| **Age**  (years) (s.d) | **41.33** (12.36) | **38.09** (14.86) | **41.55** (15.00) | **0.30** |
| **Gender *female*,**  *N (%)* | **98** (77.2) | **38** (88.4) | **60** (75.0) | **0.20** |
| **BMI**  (kgm^-2^) (s.d) | **30.90** (6.42) | **23.51** (3.91) | **24.44** (4.54) | **<0.001** |

All decimal values given to two significant figures except p-values given to one significant figure. Missing data: BMI (n=1) in infl CRP>1, BMI (n=1) in healthy controls. Abbreviations: ANOVA, analysis of variance test; BMI, body mass index; Infl CRP>1, inflammatory depression group; Non-infl CRP<1, non-inflammatory depression group.

Supplementary Table 4. **Change in biomarkers for patients (n=33) receiving *Lactobacillus reuteri* probiotic.** Mean absolute biomarker concentrations measured at baseline (BL) and end of study (week 8). Paired samples t-test was performed using logarithmically transformed values.

| **Biomarker** | **Time** | **Mean absolute concentrations**  **(μM) (s.d)** | **Paired samples t-test** | |
| --- | --- | --- | --- | --- |
|  |  |  | **Point estimate - *Cohen's d*** | **Two-sided p** |
| **NAA** | BL | 0.40 (0.17) | 0.41 | **0.025*** |
|  | w8 | 0.33 (0.16) |  |  |
| **PIC** | BL | 0.018 (0.016) | -0.30 | 0.806 |
|  | w8 | 0.017 (0.011) |  |  |
| **QUIN** | BL | 0.51 (0.21) | 0.13 | 0.444 |
|  | w8 | 0.50 (0.27) |  |  |
| **KYNA** | BL | 0.061 (0.023) | -0.18 | 0.298 |
|  | w8 | 0.061 (0.020) |  |  |
| **Trp** | BL | 43.54 (9.44) | 0.22 | 0.213 |
|  | w8 | 40.36 (7.98) |  |  |
| **Kyn** | BL | 2.61 (0.78) | 0.001 | 0.997 |
|  | w8 | 2.53 (0.63) |  |  |
| **3-HK** | BL | 0.035 (0.013) | -0.009 | 0.957 |
|  | w8 | 0.034 (0.012) |  |  |

* p<0.05; **p<0.01; ***p<0.001.

All decimal values given to two significant figures. Missing data: PA (n=1). Abbreviations: BL, baseline; KYNA, kynurenic acid; Kyn, kynurenine; NAA, nicotinamide; PIC, picolinic acid; QUIN, quinolinic acid; s.d., standard deviation; Trp, tryptophan; 3-HK, 3-hydroxykynurenine.

Supplementary Table 5. **Change in biomarkers for subjects receiving no active intervention (placebo and healthy controls) (n=44).** Mean absolute biomarker concentrations measured at baseline (BL) and end of study (week 8). Paired samples t-test was performed using logarithmically transformed values.

| **Biomarker** | **Time** | **Mean absolute concentrations**  **(μM) (s.d)** | **Paired samples t-test** | |
| --- | --- | --- | --- | --- |
|  |  |  | **Point estimate - *Cohen's d*** | **Two-sided p** |
| **NAA** | BL | 0.38 (0.17) | 0.50 | **0.002**** |
|  | w8 | 0.31 (0.15) |  |  |
| **PIC** | BL | 0.015 (0.0073) | 0.037 | 0.81 |
|  | w8 | 0.014 (0.0062) |  |  |
| **QUIN** | BL | 0.41 (0.17) | 0.098 | 0.52 |
|  | w8 | 0.43 (0.18) |  |  |
| **KYNA** | BL | 0.059 (0.021) | 0.17 | 0.25 |
|  | w8 | 0.059 (0.024) |  |  |
| **Trp** | BL | 44.50 (6.78) | 0.096 | 0.53 |
|  | w8 | 43.98 (8.76) |  |  |
| **Kyn** | BL | 2.34 (0.53) | 0.16 | 0.29 |
|  | w8 | 2.40 (0.47) |  |  |
| **3-HK** | BL | 0.029 (0.0088) | 0.25 | 0.10 |
|  | w8 | 0.029 (0.0080) |  |  |

* p<0.05; **p<0.01; ***p<0.001.

All decimal values given to two significant figures. Missing data: PA (n=1), LK (n=1). Abbreviations: BL, baseline; KYNA, kynurenic acid; Kyn, kynurenine; NAA, nicotinamide; PIC, picolinic acid; QUIN, quinolinic acid; s.d., standard deviation; Trp, tryptophan; 3-HK, 3-hydroxykynurenine.
