# Supplementary material for: Kynurenine pathway metabolites are increased in inflammatory depression and decrease with omega-3 treatment
Source: Brain Behav Immun Health. 2026 Mar 25;53:101221. doi: 10.1016/j.bbih.2026.101221 (PMC13066792; doi:10.1016/j.bbih.2026.101221)
Supplement: Multimedia component 2 [file mmc2.docx]

Supplementary Table 6. **Group-wise comparison of baseline biomarkers between inflammatory depression, non-inflammatory depression and healthy controls.** Mean absolute concentrations of biomarkers measured at baseline. Subjects stratified per baseline blood high-sensitivity C-reactive protein (hs-CRP) into inflammatory depression (hs-CRP≥3 mg/L), non-inflammatory depression (hs-CRP<3 mg/L) and healthy controls. Group-wise comparisons conducted with analysis of variance (ANOVA) tests. Post hoc Bonferroni corrected the level of statistical significance of the ANOVA tests.

| **Mean (SD) of biomarker concentrations at baseline** | | | |
| --- | --- | --- | --- |
| **Variable** | **Inflammatory depression (CRP≥3)** | **Non-inflammatory depression (CRP<3)** | **Healthy controls** |
| **Evaluated subjects**, *N* | **64** | **106** | **80** |
| **NAA** (μM) | **0.44** (0.21) | **0.40** (0.15) | **0.37** (0.15) |
| **PIC** (μM) | **0.015** (0.0091) | **0.015** (0.0101) | **0.016** (0.0079) |
| **QUIN** (μM) | **0.49** (0.19) | **0.39** (0.16) | **0.38** (0.17) |
| **KA** (μM) | **0.058** (0.022) | **0.054** (0.020) | **0.059** (0.022) |
| **Trp** (μM) | **43.19** (7.81) | **42.68** (7.27) | **44.35** (6.10) |
| **Kyn** (μM) | **2.47** (0.50) | **2.25** (0.65) | **2.27** (0.50) |
| **3-HK** (μM) | **0.033** (0.0108) | **0.027** (0.0092) | **0.028** (0.0089) |
| ***Post hoc* Bonferroni adjusted p-values are reported** | | | |
| **Biomarker** | **Groups (A)** | **Groups (B)** | **p-value** |
|  |  |  |  |
| **NAA** | Healthy controls | Non-inflammatory depression | 0.42 |
|  |  | Inflammatory depression | **0.027** |
|  | Non-inflammatory depression | Healthy controls | 0.42 |
|  |  | Inflammatory depression | 0.49 |
|  | Inflammatory depression | Healthy controls | **0.027** |
|  |  | Non-inflammatory depression | 0.49 |
| **PIC** | Healthy controls | Non-inflammatory depression | 1.00 |
|  |  | Inflammatory depression | 0.90 |
|  | Non-inflammatory depression | Healthy controls | 1.00 |
|  |  | Inflammatory depression | 1.00 |
|  | Inflammatory depression | Healthy controls | 0.90 |
|  |  | Non-inflammatory depression | 1.00 |
| **QUIN** | Healthy controls | Non-inflammatory depression | 1.00 |
|  |  | Inflammatory depression | **<0.001** |
|  | Non-inflammatory depression | Healthy controls | 1.00 |
|  |  | Inflammatory depression | **<0.001** |
|  | Inflammatory depression | Healthy controls | **<0.001** |
|  |  | Non-inflammatory depression | **<0.001** |
| **KA** | Healthy controls | Non-inflammatory depression | 0.33 |
|  |  | Inflammatory depression | 1.00 |
|  | Non-inflammatory depression | Healthy controls | 0.33 |
|  |  | Inflammatory depression | 0.78 |
|  | Inflammatory depression | Healthy controls | 1.00 |
|  |  | Non-inflammatory depression | 0.78 |
| **Trp** | Healthy controls | Non-inflammatory depression | 0.34 |
|  |  | Inflammatory depression | 0.99 |
|  | Non-inflammatory depression | Healthy controls | 0.34 |
|  |  | Inflammatory depression | 1.00 |
|  | Inflammatory depression | Healthy controls | 0.99 |
|  |  | Non-inflammatory depression | 1.00 |
| **Kyn** | Healthy controls | Non-inflammatory depression | 1.00 |
|  |  | Inflammatory depression | 0.12 |
|  | Non-inflammatory depression | Healthy controls | 1.00 |
|  |  | Inflammatory depression | 0.052 |
|  | Inflammatory depression | Healthy controls | 0.12 |
|  |  | Non-inflammatory depression | 0.052 |
| **3-HK** | Healthy controls | Non-inflammatory depression | 1.00 |
|  |  | Inflammatory depression | **0.009** |
|  | Non-inflammatory depression | Healthy controls | 1.00 |
|  |  | Inflammatory depression | **<0.001** |
|  | Inflammatory depression | Healthy controls | **0.009** |
|  |  | Non-inflammatory depression | **<0.001** |

Missing data: NAA (n=1), PA (n=6), QA (n=2), KA (n=2), Trp (n=2), LK (n=3). Abbreviations: BMI, body-mass index; Infl, inflammation; KA, kynurenic acid; Kyn, kynurenine; NAA, nicotinamide; Non-infl, non-inflammation; PA, picolinic acid; QA, quinolinic acid; s.d., standard deviation; Trp, tryptophan; 3-HK, 3-hydroxykynurenine.
