# Supplementary material for: Kynurenine pathway metabolites are increased in inflammatory depression and decrease with omega-3 treatment
Source: Brain Behav Immun Health. 2026 Mar 25;53:101221. doi: 10.1016/j.bbih.2026.101221 (PMC13066792; doi:10.1016/j.bbih.2026.101221)
Supplement: Multimedia component 3 [file mmc3.docx]

Supplementary Table 7. **Summary of significant correlations with Benjamini-Hochberg FDR-adjusted p-values.** Presentation in order of appearance in the manuscript.

| **Correlation** | **Cohort** | **p-value** | **FDR adjusted** |
| --- | --- | --- | --- |
| Baseline Trp vs baseline SHAPS | n-3 PUFA | 0.007 | **0.049** |
| Change in KYNA vs baseline HDRS-17 | n-3 PUFA | 0.042 | 0.1143333 |
| Change in Kyn vs baseline HDRS-17 | n-3 PUFA | 0.043 | 0.1143333 |
| Change in 3-HK vs baseline HDRS-17 | n-3 PUFA | 0.049 | 0.1143333 |
| Baseline QUIN in responders vs non-responders | n-3 PUFA | 0.038 | 0.0665 |
| Baseline KYNA in responders vs non-responders | n-3 PUFA | 0.003 | **0.0105** |
| Baseline Kyn in responders vs non-responders | n-3 PUFA | 0.003 | **0.0105** |
| Baseline 3-HK in responders vs non-responders | n-3 PUFA | 0.025 | 0.0583333 |
| Baseline QUIN vs change in FSS | n-3 PUFA | 0.023 | 0.0536667 |
| Baseline QUIN vs change in ISI | n-3 PUFA | 0.033 | 0.08225 |
| Baseline KYNA vs change in FSS | n-3 PUFA | 0.003 | **0.021** |
| Baseline KYNA vs change in GAD-7 | n-3 PUFA | 0.049 | 0.343 |
| Baseline KYNA vs change in ISI | n-3 PUFA | 0.047 | 0.08225 |
| Baseline Kyn vs change in FSS | n-3 PUFA | 0.039 | 0.06825 |
| Baseline Kyn vs change in ISI | n-3 PUFA | 0.005 | **0.035** |
| Baseline 3-HK vs change in FSS | n-3 PUFA | 0.012 | **0.042** |
| Baseline 3-HK vs change in ISI | n-3 PUFA | 0.042 | 0.08225 |
| Baseline QUIN vs change in inflammatory depression composite score | Probiotics | 0.018 | 0.126 |
| Baseline QUIN vs change in PHQ-9 | Probiotics | 0.027 | 0.112 |
| Baseline Kyn vs change in PHQ-9 | Probiotics | 0.032 | 0.112 |
| Change in QUIN among n-3 PUFA treated | n-3 PUFA | 0.017 | **0.0396667** |
| Change in KYNA among n-3 PUFA treated | n-3 PUFA | 0.006 | **0.0396667** |
| Change in 3-HK among n-3 PUFA treated | n-3 PUFA | 0.016 | **0.0396667** |
| Change in Trp vs change in SHAPS | n-3 PUFA | 0.043 | 0.231 |
| Change in QUIN among SHAPS responders | n-3 PUFA | 0.010 | 0.07 |
| Change in 3-HK among SHAPS responders | n-3 PUFA | 0.003 | **0.042** |
| Change in QUIN SHAPS responders vs non-responders | n-3 PUFA | 0.046 | 0.217 |
| Change in QUIN vs change in FSS | n-3 PUFA | 0.025 | 0.14 |
| Change in 3-HK vs change in FSS | n-3 PUFA | 0.040 | 0.14 |
| Change in NAA among probiotics treated | Probiotics | 0.025 | 0.175 |
| Change in NAA among healthy controls and placebo treated | Probiotics | 0.002 | **0.014** |
| Change in QUIN vs change in MADRS-M | Probiotics | 0.031 | 0.217 |

Abbrevations: FSS, Fatigue Severity Scale; GAD-7, Generalized Anxiety Disorder 7-item scale; HDRS-17, Hamilton Depression Rating Scale 17-item; ISI, Insomnia Severity Index; KA, kynurenic acid; Kyn, kynurenine; MADRS-M, Montgomery-Åsberg Depression Rating Scale, NAA, nicotinamide; n-3 PUFAs, omega-3 polyunsaturated fatty acids; PHQ-9, Patient Health Questionnaire-9; PIC, picolinic acid; QUIN, quinolinic acid; SHAPS, Snaith-Hamilton Pleasure Scale; Trp, tryptophan; 3-HK, 3-hydroxykynurenine.
