# Supplementary material for: Kynurenine pathway metabolites are increased in inflammatory depression and decrease with omega-3 treatment
Source: Brain Behav Immun Health. 2026 Mar 25;53:101221. doi: 10.1016/j.bbih.2026.101221 (PMC13066792; doi:10.1016/j.bbih.2026.101221)
Supplement: Multimedia component 4 [file mmc4.docx]

Supplementary Tables 8-9 to ”Kynurenine pathway metabolites are increased in inflammatory depression and decrease with omega-3 treatment”

Supplementary Table 8. **Spearman’s rank order correlations between KP metabolites/biomarkers (baseline and delta values) and rating scale changes from baseline to week 8 (primary and secondary treatment response outcome measures) among the patients with n-3 PUFA treatment.**

| **Biomarker** |  | **Δ HDRS-17** | **Δ FSS** | **Δ ISI** | **Δ GAD-7** | **Δ PHQ-9** | **Δ SHAPS** |
| --- | --- | --- | --- | --- | --- | --- | --- |
| BL Trp | Correlation Coefficient | .027 | .004 | .095 | -.032 | .065 | .092 |
|  | Sig. (2-tailed) | .806 | .973 | .380 | .765 | .550 | .392 |
|  | N | 88 | 88 | 88 | 88 | 88 | 88 |
| Δ Trp | Correlation Coefficient | -.170 | -.132 | -.135 | .057 | -.034 | -.192 |
|  | Sig. (2-tailed) | .117 | .225 | .214 | .604 | .759 | .076 |
|  | N | 86 | 86 | 86 | 86 | 86 | 86 |
| BL Kyn | Correlation Coefficient | -.216 | -.221 | -.294 | -.079 | -.131 | .100 |
|  | Sig. (2-tailed) | **.043** | **.039** | **.005** | .465 | .224 | .356 |
|  | N | 88 | 88 | 88 | 88 | 88 | 88 |
| Δ Kyn | Correlation Coefficient | .088 | -.006 | -.030 | .008 | .019 | -.005 |
|  | Sig. (2-tailed) | .420 | .959 | .787 | .944 | .863 | .961 |
|  | N | 86 | 86 | 86 | 86 | 86 | 86 |
| BL KYNA | Correlation Coefficient | -.217 | -.317 | -.212 | -.211 | -.168 | .038 |
|  | Sig. (2-tailed) | **.042** | **.003** | **.047** | **.049** | .117 | .728 |
|  | N | 88 | 88 | 88 | 88 | 88 | 88 |
| Δ KYNA | Correlation Coefficient | -.001 | .185 | .021 | .100 | -.014 | -.082 |
|  | Sig. (2-tailed) | .990 | .088 | .846 | .357 | .897 | .451 |
|  | N | 86 | 86 | 86 | 86 | 86 | 86 |
| BL QUIN | Correlation Coefficient | -.159 | -.242 | -.228 | -.027 | -.114 | .023 |
|  | Sig. (2-tailed) | .140 | **.023** | **.033** | .800 | .289 | .830 |
|  | N | 88 | 88 | 88 | 88 | 88 | 88 |
| Δ QUIN | Correlation Coefficient | .084 | .242 | -.080 | -.080 | -.001 | .062 |
|  | Sig. (2-tailed) | .442 | **.025** | .466 | .464 | .994 | .573 |
|  | N | 86 | 86 | 86 | 86 | 86 | 86 |
| BL PIC | Correlation Coefficient | -.057 | .031 | -.032 | -.091 | -.044 | -.180 |
|  | Sig. (2-tailed) | .600 | .779 | .770 | .403 | .686 | .096 |
|  | N | 87 | 87 | 87 | 87 | 87 | 87 |
| Δ PIC | Correlation Coefficient | -.199 | -.086 | -.083 | -.050 | -.182 | -.032 |
|  | Sig. (2-tailed) | .071 | .441 | .454 | .654 | .100 | .774 |
|  | N | 83 | 83 | 83 | 83 | 83 | 83 |
| BL 3-HK | Correlation Coefficient | -.208 | -.265 | -.214 | -.073 | -.122 | .052 |
|  | Sig. (2-tailed) | **.049** | **.012** | **.042** | .492 | .253 | .626 |
|  | N | 90 | 90 | 90 | 90 | 90 | 90 |
| Δ 3-HK | Correlation Coefficient | .100 | .219 | .058 | -.045 | .011 | .105 |
|  | Sig. (2-tailed) | .354 | **.040** | .589 | .678 | .922 | .329 |
|  | N | 88 | 88 | 88 | 88 | 88 | 88 |
| BL NAA | Correlation Coefficient | .057 | .154 | .086 | -.051 | .104 | .010 |
|  | Sig. (2-tailed) | .596 | .150 | .422 | .638 | .334 | .925 |
|  | N | 89 | 89 | 89 | 89 | 89 | 89 |
| Δ NAA | Correlation Coefficient | -.069 | -.151 | .160 | -.105 | .064 | -.078 |
|  | Sig. (2-tailed) | .527 | .164 | .139 | .335 | .554 | .474 |
|  | N | 87 | 87 | 87 | 87 | 87 | 87 |

Abbreviations: BL, baseline; Δ, delta, change from baseline to week 8 follow-up; HDRS-17, Hamilton Depression Rating Scale 17-item; FSS, Fatigue Severity Scale; ISI, Insomnia Severity Index; GAD-7, Generalized Anxiety Disorder 7-item scale; PHQ-9, Patient Health Questionnaire-9; SHAPS, Snaith-Hamilton Pleasure Scale; Trp, tryptophan; Kyn, kynurenine; KYNA, kynurenic acid; QUIN, quinolinic acid; PIC, picolinic acid; 3-HK, 3-hydroxykynurenine; NAA, nicotinamide

Supplementary Table 9. **Spearman’s rank order correlations between KP metabolites/biomarkers (baseline and delta values) and rating scale changes from baseline to week 8 (primary and secondary treatment response outcome measures) among the patients with active probiotics treatment.**

| **Biomarker** |  | **Δ MADRS-M** | **Δ FSS** | **Δ ISI** | **Δ GAD-7** | **Δ PHQ-9** |
| --- | --- | --- | --- | --- | --- | --- |
| BL Trp | Correlation Coefficient | -.135 | .166 | -.128 | -.015 | .235 |
|  | Sig. (2-tailed) | .426 | .347 | .472 | .933 | .181 |
|  | N | 37 | 34 | 34 | 34 | 34 |
| Δ Trp | Correlation Coefficient | .286 | -.105 | .294 | .048 | .009 |
|  | Sig. (2-tailed) | .106 | .574 | .109 | .799 | .962 |
|  | N | 33 | 31 | 31 | 31 | 31 |
| BL Kyn | Correlation Coefficient | .082 | .081 | .072 | .178 | .368 |
|  | Sig. (2-tailed) | .628 | .649 | .686 | .313 | **.032** |
|  | N | 37 | 34 | 34 | 34 | 34 |
| Δ Kyn | Correlation Coefficient | -.003 | -.193 | .011 | -.072 | -.103 |
|  | Sig. (2-tailed) | .986 | .299 | .952 | .701 | .583 |
|  | N | 33 | 31 | 31 | 31 | 31 |
| BL KYNA | Correlation Coefficient | -.025 | -.006 | -.002 | .249 | .136 |
|  | Sig. (2-tailed) | .881 | .972 | .991 | .155 | .442 |
|  | N | 37 | 34 | 34 | 34 | 34 |
| Δ KYNA | Correlation Coefficient | -.062 | .089 | .124 | -.145 | -.129 |
|  | Sig. (2-tailed) | .731 | .635 | .507 | .436 | .489 |
|  | N | 33 | 31 | 31 | 31 | 31 |
| BL QUIN | Correlation Coefficient | .211 | .109 | .283 | .330 | .380 |
|  | Sig. (2-tailed) | .210 | .538 | .105 | .057 | **.027** |
|  | N | 37 | 34 | 34 | 34 | 34 |
| Δ QUIN | Correlation Coefficient | -.376 | -.077 | -.156 | -.321 | -.283 |
|  | Sig. (2-tailed) | **.031** | .681 | .402 | .079 | .123 |
|  | N | 33 | 31 | 31 | 31 | 31 |
| BL PIC | Correlation Coefficient | .006 | .107 | -.120 | .109 | .104 |
|  | Sig. (2-tailed) | .970 | .552 | .507 | .546 | .564 |
|  | N | 36 | 33 | 33 | 33 | 33 |
| Δ PIC | Correlation Coefficient | -.181 | .062 | .083 | -.150 | -.106 |
|  | Sig. (2-tailed) | .321 | .743 | .664 | .430 | .577 |
|  | N | 32 | 30 | 30 | 30 | 30 |
| BL 3-HK | Correlation Coefficient | .173 | .118 | .199 | .282 | .255 |
|  | Sig. (2-tailed) | .306 | .506 | .260 | .106 | .145 |
|  | N | 37 | 34 | 34 | 34 | 34 |
| Δ 3-HK | Correlation Coefficient | .062 | -.051 | .014 | .018 | .061 |
|  | Sig. (2-tailed) | .731 | .787 | .941 | .922 | .745 |
|  | N | 33 | 31 | 31 | 31 | 31 |
| BL NAA | Correlation Coefficient | .238 | .234 | .016 | .094 | .238 |
|  | Sig. (2-tailed) | .156 | .184 | .929 | .598 | .175 |
|  | N | 37 | 34 | 34 | 34 | 34 |
| Δ NAA | Correlation Coefficient | .066 | -.021 | -.117 | .052 | -.031 |
|  | Sig. (2-tailed) | .716 | .909 | .530 | .783 | .867 |
|  | N | 33 | 31 | 31 | 31 | 31 |

Abbreviations: BL, baseline; Δ, delta, change from baseline to week 8 follow-up; MADRS-M, Montgomery-Åsberg Depression Rating Scale; FSS, Fatigue Severity Scale; ISI, Insomnia Severity Index; GAD-7, Generalized Anxiety Disorder 7-item scale; PHQ-9, Patient Health Questionnaire-9; Trp, tryptophan; Kyn, kynurenine; KYNA, kynurenic acid; QUIN, quinolinic acid; PIC, picolinic acid; 3-HK, 3-hydroxykynurenine; NAA, nicotinamide
